# Sensitivity and specificity of OraQuick® HIV self-test compared to a 4th generation laboratory reference standard algorithm in urban and rural Zambia

**DOI:** 10.1186/s12879-022-07457-5

**Published:** 2022-05-25

**Authors:** Melissa Neuman, Alwyn Mwinga, Kezia Kapaku, Lucheka Sigande, Caroline Gotsche, Miriam Taegtmeyer, Russell Dacombe, Kwitaka Maluzi, Barry Kosloff, Cheryl Johnson, Karin Hatzold, Elizabeth L. Corbett, Helen Ayles

**Affiliations:** 1 MRC International Statistics and Epidemiology Group and Department of Infectious Disease Epidemiology, London School of Hygiene and Tropical Medicine, London, UK; 2 Zambart, University of Zambia School of Public Health, Ridgeway Campus, Off Nationalist Road, Lusaka, Zambia; 3 London School of Hygiene and Tropical Medicine, Keppel Street, London, WC1E 7HT UK; 4 Liverpool School of Tropical Medicine, Pembroke Pl, Liverpool, L3 5QA UK; 5 Tropical Infectious Diseases Unit, Royal Liverpool University Hospital, Liverpool, L7 8XP UK; 6 World Health Organization, 20 Avenue Appia, Geneva, Switzerland; 7 PSI-South Africa, 70, 7th Avenue, Rosebank, Johannesburg, South Africa; 8 Malawi-Liverpool-Wellcome Trust Clinical Research Unit, Blantyre, Malawi

**Keywords:** HIV self-testing, HIV, Diagnostic tests, In vitro diagnostics, Self-care

## Abstract

**Background:**

HIV self-testing (HIVST) has the potential to increase coverage of HIV testing, but concerns exist about intended users’ ability to correctly perform and interpret tests, especially in poor communities with low literacy rates. We assessed the clinical performance of the 2016 prototype OraQuick® HIV Self-Test in rural and urban communities in Zambia to assess the sensitivity and specificity of the test compared to the national HIV rapid diagnostic test (RDT) algorithm and a laboratory reference standard using 4th generation enzyme immunoassays and HIV RNA detection.

**Methods:**

Participants were recruited from randomly selected rural and urban households and one urban health facility between May 2016 and June 2017. Participants received a brief demonstration of the self-test, and then self-tested without further assistance. The research team re-read the self-test, repeated the self-test, drew blood for the laboratory reference, and conducted RDTs following the national HIV testing algorithm (Determine™ HIV1/2 (Alere) confirmed using Unigold™ HIV1/2 (Trinity Biotech)). Selected participants (N = 85) were videotaped whilst conducting the testing to observe common errors.

**Results:**

Initial piloting showed that written instructions alone were inadequate, and a demonstration of self-test use was required. Of 2,566 self-test users, 2,557 (99.6%) were able to interpret their result. Of participants who were videoed 75/84 (89.3%) completed all steps of the procedure correctly.

Agreement between the user-read result and the researcher-read result was 99.1%. Compared to the RDT algorithm, user-conducted HIVST was 94.1% sensitive (95%CI: 90.2–96.7) and 99.7% specific (95%CI: 99.3–99.9). Compared to the laboratory reference, both user-conducted HIVST (sensitivity 87.5%, 95%CI: 82.70–91.3; specificity 99.7%, 95%CI: 99.4–99.9) and the national RDT algorithm (sensitivity 93.4%, 95%CI: 89.7–96.1%; specificity 100% (95%CI: 99.8–100%) had considerably lower sensitivity.

**Conclusions:**

Self-testers in Zambia who used OraQuick® HIV Self-Test achieved reasonable clinical performance compared to the national RDT algorithm. However, sensitivity of the self-test was reduced compared to a laboratory reference standard, as was the national RDT algorithm. In-person demonstration, along with the written manufacturer instructions, was needed to obtain accurate results. Programmes introducing self-care diagnostics should pilot and optimise support materials to ensure they are appropriately adapted to context.

**Supplementary Information:**

The online version contains supplementary material available at 10.1186/s12879-022-07457-5.

## Background

For the post-Millennium Development Goals era, the United Nations set ambitious testing and treatment goals to reach and maintain low HIV incidence by 2030. Meeting these targets requires more concerted efforts to improve access to, and acceptance of, HIV testing.

HIV self-testing (HIVST), whereby an individual conducts an HIV rapid diagnostic test (RDT) using either fingerstick/whole blood or oral fluid specimen and interprets their own result, is an important strategy to increase access to and uptake of HIV testing services (HTS). HIVST is recommended by the World Health Organization as a “test for triage” [1]. HIVST has the potential to provide early diagnosis by removing barriers to HIV testing, and to allow individuals at highest risk of HIV to access HIV prevention.

HIVST is safe, acceptable and effective for increasing uptake, frequency and coverage of testing in many populations and settings [2–4], but assessments of the reliability and validity of HIVST show more mixed results. A 2018 systematic review of studies of the accuracy of HIVST conducted prior to WHO pre-qualification of any HIVST showed good concordance between intended-user and professional-user, but a wide range of sensitivity from 66 to 99.1% [5]. Likely causes of heterogeneity included differences in the study populations and settings, including level of support provided to self-testers, and reference standard tests which varied from laboratory-based tests to fingerprick HIV RDTs. Most (11/14) studies in this systematic review used prototypes of the OraQuick® Advance HIV1/2, which was in the process of being adapted for HIVST.

Despite substantial progress in testing coverage over the past decade, prevalence of HIV in Zambia remains high, with substantial gaps in testing uptake. The 2016 Zambia Population HIV Impact Assessment found HIV prevalence among 15 to 59 year olds to be 14.9% for women and 9.5% for men, with only 67.3% of persons living with HIV (PLHIV) aware of their status [6]. Significant barriers to accessing facility-based HIV testing services include both structural barriers, such as the direct and opportunity costs associated with seeking HIV testing services at a health facility, and individual-level barriers, including fear of confidentiality of testing and a low perceived risk of HIV infection. HIVST has great potential to overcome these barriers and reduce testing gaps [7]. However, to ensure HIVST are successful at reaching and screening all persons at risk of HIV acquisition, it is essential for programme implementers and regulators to understand whether the test can be used by people from a variety of backgrounds.

The main aim of this study was to investigate the clinical performance (i.e. sensitivity and specificity) of OraQuick® HIV Self-Test (Orasure Technologies LLC, Thailand) in an untrained population of intended self-testers, compared with the Zambian national algorithm using RDTs and also a laboratory-based testing algorithm using 4^th^ generation enzyme immune-assay (EIA) comparators. Since previous studies have suggested that rural users or users with lower literacy levels may have more challenges with testing correctly [8], and home settings are different from clinical settings, we aimed to recruit intended users in their homes in both rural and urban settings as well as in a health facility. The study had 3 components: (1) we first conducted a pilot study to understand how much guidance users would need to complete the study; (2) we completed the main study of sensitivity and specificity of HIVST against both the laboratory assessment and the RDT algorithm, including assessments of test performance by age, place of recruitment, and educational attainment; (3) a sub-sample within the main study were video recorded to understand common performance and interpretation errors in order to optimize future demonstrations, messages and instructions. We also report on the sensitivity and specificity of the Zambian RDT algorithm against the laboratory standard.

## Methods

### Overall study design and recruitment

This study used a cross-sectional design, and both study and analysis were conducted in accordance with STARD (Standards for Reporting Diagnostic Accuracy Studies) guidelines for reporting diagnostic accuracy studies [9]. Consenting adolescent and adult participants (≥ 15 years) were recruited from two sites in urban and rural Lusaka Province, Zambia. Adult respondents were asked to provide informed consent; adolescent participants provided assent and parental consent for participation. Locations for study data collection were chosen to provide access to intended user populations and also to be in close proximity to the study laboratory, so blood samples could be processed in a timely manner.

To select household participants, sample enumeration areas (SEAs) were identified using Census data, and chosen at random. Within each randomly selected SEA, all households were visited following community sensitization. Additional urban participants were recruited from the HIV testing services of a primary care clinic offering voluntary counselling and testing services. All persons attending the facility for voluntary HIV testing services facility attendees fitting the inclusion criteria were approached and asked to participate in the study.

Participants who self-reported themselves to be HIV-positive and taking anti-retroviral therapy (ART) were excluded from the analysis of sensitivity and specificity.

### Main clinical performance study

The main clinical performance study was conducted from June 2016-June 2017. A research assistant (RA) administered a standardised questionnaire to collect data on participant socio-demographics and HIV testing history. After this, the RA talked participants through a brief demonstration (approximately 10 min) of the testing steps following a pre-defined training checklist (Additional file 1: Table S1). Participants were asked to test themselves in private with an OraQuick® HIV Self-Test kit including the manufacturer’s IFU, using a self-collected oral fluid specimen. Participants recorded their test result as either reactive, non-reactive, or invalid on a self-assessment questionnaire (“user-conducted user-read” test result). They were also asked to report any errors they made during testing. The completed self-assessment questionnaire and used test kit were returned to the research assistant (RA) who immediately reread the user’s self-test kit and recorded the result (“user-conducted researcher-read”) before comparing this with the participant’s self-completed questionnaire. The RA repeated the OraQuick® Self-Test on the participant using a fresh test kit and recorded the result.

Research nurses blind to the self-test results conducted rapid HIV testing (Determine™ HIV1/2 (Alere) confirmed, if reactive, using Unigold™ HIV1/2 (Trinity Biotech)) as per the national serial testing algorithm. They also drew venous blood for further laboratory testing. Participants were provided with post-test counselling and referral to care and support services if HIV-positive according to the national RDT algorithm.

The results of the self-test, as performed and read by the intended user, was compared to the same test performed and read by the RA. The self-test by the intended user was then compared to the result of the RDT algorithm and the laboratory reference standard described below. The field-based RDT algorithm was also compared to the laboratory reference standard.

#### Laboratory HIV testing procedure

Venous blood (10 mls) collected into EDTA was processed within 8 h at the Zambart central laboratory. This laboratory was registered under the College of American Pathologists (CAP) External Quality Assurance (EQA) programme for the Abbott Architect HIV Ag/Ab Combo assay and a certified laboratory by the National Institutes of Health HIV Prevention Trials Network (HPTN).

The whole blood was centrifuged at 800 × *g* for 10 min and plasma stored at − 80 °C. All plasma samples were tested according to a composite reference standard algorithm as shown in Additional file 1: Fig. S1 (Laboratory Reference Standard).

All samples were tested using the Abbott Architect HIV1 Ag/Ab combo EIA test. Reactive tests on this assay were confirmed using a second 4th generation assay, Bio-Rad GS HIV Combo Ag/Ab assay (Bio-Rad, Hercules, CA, USA). Any discrepant results were retested using Geenius and HIV RNA testing (Additional file 1: Fig. S1). Cut-off points and interpretation criteria for all laboratory tests were as specified by the manufacturer. On samples with discrepant results, we additionally conducted viral load testing and RDT testing using plasma was rather than whole blood.

Laboratory reference standard results were not provided to study participants. Laboratory testers were not blinded to the OraQuick® HIV Self-Test or RDT results of the study participants.

#### Sample size calculation

The study size was calculated assuming the true HIV prevalence in the tested population was 12.5%, and the true sensitivity and specificity of the test were 93.0% and 99.9%, respectively, against national RDT algorithm [10]. The target sample size was 3,209 (400 HIV positive) to provide precision around exact binomial 95% confidence intervals of 90.1–95.1% for sensitivity, and 99.6–99.9% for specificity. Because of constraints on time for recruiting participants into the study, the sample size was recalculated after 6 months of recruitment using measured HIV prevalence from recruited respondents. The revised target sample size was 2,200 (216 HIV positive) to provide precision around 95% confidence intervals of 89.0%-95.7% for sensitivity and 99.7%-100% for specificity.

### Pilot study

A pilot study was conducted from May to June 2016 during which participants were provided with the OraQuick® HIVST, including the manufacturer-provided Information for Use (IFU) leaflet translated into the local language with no additional instructions. Testing and data collection procedures were the same as in the main clinical performance study (described above). Consenting participants were videoed to observe their performance of the test. Following review of data (see “Results” Section) a standard presentation and in-person demonstration was provided to all participants before self-testing.

### Video-recording sub-study

To assess common errors, participants were asked to consent to video recording. As part of the consent process for the video study, participants were informed that they would be recorded, and recordings observed by a health worker. Only a convenience sub-set could be videoed in practice, both because not all participants consented to videoing and because the number of video cameras available for the study was limited.

Videos were reviewed for errors by CG after the end of data collection according to standardised checklist (Additional file 1: Table S2). Conducting “all testing steps correctly” was defined as completing the process starting with the opening of the pouches until putting the flat pad into developer solution, but not the waiting period (20 min) or reading of results [10].

### Statistical analysis procedures

#### Pilot and main clinical performance study

For the pilot and main clinical performance study, we have presented sensitivity, specificity, disagreement rate and a Kappa statistic to compare participants’ user-test user-read HIVST results (reactive, non-reactive, and invalid) against 1) trained reader interpretation of participants’ self-test kits (researcher-read) and 2) the repeat OraQuick® test read by the trained-user (researcher-conducted). We have also presented sensitivity, specificity, disagreement rate and a Kappa statistic to compare participants’ results with results generated using 1) national RDT-based algorithm and 2) pre-defined laboratory reference standards.

A pre-specified subgroup analysis estimated sensitivity and specificity by participant characteristics such as sex, age, and recruitment location (rural community, urban community, urban health facility). A sensitivity analysis restricting sensitivity and specificity analyses to persons not reporting HIV positive status is included in supplementary material (Additional file 1: Table S3).

Invalid OraQuick®, invalid or inconclusive/discrepant RDT results, and respondents with missing data were not included in calculations of specificity, sensitivity, percent disagreement, or Kappa, with numbers of missing respondents indicated in the table footnotes. There were no inconclusive laboratory test results.

#### Video sub-study

In the video sub-study, we calculated the proportion of respondents in the study completing each component of self-test process, and used Pearson’s chi-squared tests to assess differences between sub-groups.

All analyses were completed in Stata 15.1 (College Station, TX, USA). The *diagt* [11] and *kappaetc* [12] commands were used to calculate diagnostic accuracy and Kappa statistic.

## Results

We recruited 76 participants into the pilot and 2,572 into the main clinical performance study.

### Pilot study

We first evaluated performance in a pilot where participants were provided with manufacturer’s IFU translated into local languages but no additional demonstration or other materials. Other than the lack of demonstration, procedures for the pilot were otherwise the same as the main clinical performance study.

Out of 76 participants, 2 did not complete the self-testing procedure and 8/74 (10.8%) recorded invalid or uninterpretable results. One respondent did not have an RDT result. In the remaining 65 participants (5 HIV-positive), sensitivity compared with the national RDT diagnostic algorithm was 40.0% (95% CI: 5.3%-85.3%) and specificity was 98.3% (95% CI: 91.1%-100.0%). Two participants self-reported not waiting for the full 20 min before reading their result.

Of 76 pilot participants, 3 (3.9%) were also videoed. Review of three video recorded participants during the pilot showed multiple major errors consistent with the very low accuracy, including swabbing the wrong body part, being unable to place the developer fluid vial into the stand, and not recognising the need to place the test kit into the vial and wait for 20 min.

Following these results an in-person demonstration protocol was developed using standardized demonstrations of the steps involved in self-testing evaluated on video until the study team were satisfied that participants would be able to conduct the tests correctly.

### Main clinical performance study

**Fig. 1:**
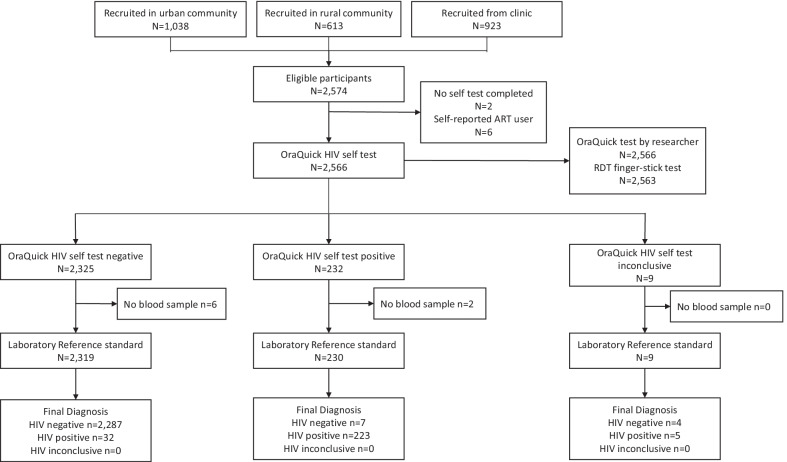
STARD diagram of flow through study

We recruited 2,574 participants for the main clinical performance study. We recruited 613 rural participants (23.8%) from 311 rural households, and 1,038 urban community participants (40.3%) from 732 urban households. 923 participants (35.8%) were recruited from the urban health facility. Over half of all participants (59.4%; 1,529) were female, and most (93.7%; 2,413) were able to read a letter or newspaper. 8.4% (211) of the population surveyed, and 15.7% of rural participants, had not completed primary education. Previous HIV testing was reported by 85.6%, with 49.8% having tested within the past 12 months and 17 (2.1%) had previously tested HIV-positive including 6 already on ART (Table 1; Fig. 1).

**Table 1:** Summary of participant characteristics (sex, age, educational attainment, literacy, HIV testing history) by study location

	Rural community		Urban community		Urban health facility		Total	
	No	%	No	%	No	%	No	%
Total participants	613	100	1038	100	923	100	2,574	100
Female (No./% participants)	289	47.1	744	71.7	496	53.7	1529	59.4
Age (median/IQR)	31	(22, 43)	25	(20, 32)	25	(21, 32)	26	(21, 35)
Age (years) (No./% participants)								
15–17 years	30	4.9	66	6.4	12	1.3	108	4.2
18–24 years	164	26.8	438	42.2	425	46.0	1027	39.9
25–34 years	166	27.1	307	29.6	309	33.5	782	30.4
35–44 years	113	18.4	125	12.0	135	14.6	373	14.5
45–54 years	55	9.0	52	5.0	32	3.5	139	5.4
55 years and older	85	13.9	50	4.8	10	1.1	145	5.6
Educational attainment (No./% participants)								
Incomplete primary education	92	16.1	75	7.3	44	4.8	211	8.4
Complete primary education	178	31.1	182	17.8	111	12.1	471	18.7
Secondary or higher education	303	52.9	765	74.9	764	83.1	1832	72.9
Literacy: able to read a newspaper or letter (No./% participants)	514	83.8	993	95.7	906	98.2	2413	93.7
Previously tested for HIV (No./% participants)	502	81.9	882	85.0	818	88.6	2202	85.5
Tested for HIV within past 12 months (No./% participants)	261	42.6	519	50.0	501	54.4	1281	49.8
Self-reported HIV + (No./% previous testers)*	17	3.5	11	1.3	17	2.1	45	2.1
Current ART use (No./% HIV +)	2	14.3	1	10	3	17.6	6	14.6
HIV positive (based on rapid diagnostic test)(No./% with RDT results)**	40	6.5	82	7.9	124	13.6	246	9.6

*Of 2291 respondents reporting having ever tested for HIV, 42 (1.9%) did not report their HIV status

**Respondents with indeterminate RDT results are included in the denominator of this measure

#### Readability of results

Comparisons of user-read results with researcher-read and researcher-conducted OraQuick® HIV self-test results are shown in Table 2. Of 2,566 respondents completing an HIVST, 2,557 (99.6%) had recorded a result. The user and researcher interpretation disagreed for 16 test kits (99.1% agreement; kappa = 0.9501; 95% CI: 0.9295–0.9706; Table 2). To investigate whether users had conducted the test correctly, researchers conducted a second OraQuick® HIV Self-Test and read the results. Results of user-conducted and researcher-conducted oral-fluid tests had 98.9% agreement (kappa = 0.9383, 95% CI: 0.9154, 0.9612; Table 2), and 18 participants had a different result when comparing user-test researcher-read and researcher-conducted kits.

**Table 2:** Comparison of user-conducted user-read with user-conducted researcher-read and researcher-conducted OraQuick® results

	User-read			Total*	Researcher-conducted			Total*
User-read	Reactive	Non-reactive	Invalid		Reactive	Non-reactive	Invalid	
Reactive	225	7	0	232	225	7	0	232
Non-reactive	9	2315	1	2325	11	2314	0	2,325
Not sure/don't know	5	0	4	9	5	4	0	9
Total	239	2322	5	2566	247	2325	0	2,566
Agreement (%)	99.14				Agreement (%)	98.91		
Cohen's kappa	0.9501 (95% CI: 0.9295–0.9706)				Cohen's kappa	0.9383 (95% CI: 0.9154–0.9612)		

^*^ Excludes 6 self-reported ART users and 2 users missing OraQuick® results (8 total)

#### Sensitivity and specificity of oral-fluid test compared with national rapid-test algorithm and laboratory testing algorithm

Sensitivity and specificity of user-conducted user-read HIVST as measured by the two algorithms (national testing RDTs, and 4th generation laboratory reference standard algorithm) are shown in Table 3, with users who read their self-test as invalid, users who did not know their result after testing, and self-reported ART users excluded from both sensitivity and specificity calculations. A further 5 users with inclusive results on RDT were excluded from all analyses using blood-based RDT results.

**Table 3:** Agreement between user-conducted and user-read OraQuick® result and RDT Algorithm and Laboratory Reference Standard

User-read	Rapid diagnostic test (RDT) algorithm result*			Laboratory reference standard result*		
	Positive	Negative	Total**	Positive	Negative	Total***
Reactive	222	8	230	223	7	230
Non-reactive	14	2305	2,319	32	2287	2319
Sub-total	236	2313	2549	255	2294	2549
Agreement (%) Cohen's kappa	99.14 0.9481 (95% CI: 0.9265–0.9697)			Agreement (%) Cohen's kappa	98.50 0.9114 (95% CI: 0.8838- 0.9390)	
Sensitivity (%)	94.1 (95% CI: 90.2–96.7)			Sensitivity (%)	87.5 (95% CI: 82.7–91.3)	
Specificity (%)	99.7(95% CI: 99.3–99.9)			Specificity (%)	99.7 (95% CI: 99.4–99.9)	

^*^The performance of the RDT Algorithm compared to Laboratory Reference Standard was: sensitivity 93.4% (95% CI: 89.7–96.1) and specificity 100% (95% CI: 99.8–100) when compared with the laboratory gold standard

**Excludes 6 self-reported ART users, 2 users missing OraQuick® results, 5 clients missing RDT results, 9 OraQuick® results read as invalid by client, 5 clients with indeterminate RDT results (25 total; 2 clients missing both RDT and OraQuick® results)

***Excludes 6 self-reported ART users, 9 OraQuick® results read as invalid by client, 10 clients missing laboratory results, and 2 clients missing laboratory and OraQuick® results (25 total; 2 clients missing both laboratory and OraQuick® results)

The user-conducted user-read HIVST was 94.1% sensitive (95% CI: 90.2–96.7) and 99.7% specific (95% CI: 99.3–99.9) compared with the Zambian national RDT algorithm, and 87.5% sensitive (95% CI: 82.7–91.3) and 99.7% specific (95% CI: 99.4–99.9) compared with the laboratory reference standard (Table 3).

We also assessed the accuracy of the national testing RDT algorithm compared with the laboratory diagnostic algorithm. The RDT was 93.4% sensitive (95% CI: 89.7–96.1) and 100% specific (95% CI: 99.8–100) when compared with the laboratory reference standard.

17 respondents had non-reactive results based on the RDT algorithm and a positive result using laboratory testing. (Additional file 1: Table S4 and S5 for detailed breakdown of discordant results between RDT and laboratory reference). All but two cases had undetectable viral load, possibly indicating undisclosed ART use, and most had low signal to cut-off ratios on Architect testing indicating low levels of antibodies. RDTs repeated in the laboratory on plasma rather than whole blood did show weak reactive or reactive tests in 10 cases which also provides evidence of low levels of antibodies that may not be detected in fingerstick whole blood testing or oral fluid specimens.

#### Associations between participant characteristics and HIVST accuracy

User characteristics were investigated because of their high potential to affect ability to correctly follow or read HIVST instructions (Table 4).

**Table 4:** Sensitivity and sensitivity of user-read OraQuick® HIVST compared with National RDT and laboratory reference

			National RDT Reference			Laboratory Reference			
	N*	Sensitivity	95% CI	Specificity	95% CI	Sensitivity	95% CI	Specificity	95% CI
All participants	2549	94.1	(90.2–96.7)	99.7	(99.3–99.9)	87.5	(82.7, 91.3)	99.7	(99.4, 99.9)
Gender									
Men only	1037	97.1	(90.1–99.7)	99.6	(98.9–99.9)	87.2	(77.7, 93.7)	99.6	(98.9, 99.9)
Women only	1512	92.8	(87.7–96.2)	99.7	(99.2–99.9)	87.6	(81.8, 92.0)	99.8	(99.3, 100)
Location of interview									
Rural community	603	94.6	(81.8–99.3)	98.9	(97.7–99.6)	76.1	(61.2, 87.4)	98.9	(97.7, 99.6)
Urban community	1030	90.0	(81.2–95.6)	99.9	(99.4–100)	87.8	(79.0, 94.1)	100	(99.6, 100)
Urban health facility	916	96.6	(91.6–99.1)	99.9	(99.3–100)	91.3	(85.0, 95.6)	99.9	(99.3, 100)
Educational attainment									
Incomplete primary education	210	95.8	(78.9–99.9)	100	(98.0–100)	80.0	(61.4, 92.3)	100	(98.0, 100)
Completed primary education and higher	2339	93.9	(89.7–96.7)	99.6	(99.3–99.8)	88.4	(83.5, 92.3)	99.7	(99.3, 99.9)
Status known									
Only respondents not self-reporting HIV-positive status	2508	93.4	(89.0–96.5)	99.7	(99.3–99.9)	85.7	(80.3, 90.1)	99.7	(99.4, 99.9)

*Numbers shown relate to Laboratory Reference Standard, data not shown for RDT standard as denominators vary by a maximum of 3 participants. N = 2549 for all participants in the comparison with national RDT diagnostic algorithm

The sensitivity of the test was low in rural community participants when compared with the laboratory standard (sensitivity compared to laboratory reference 76.6%, 95% CI 62.0–87.7%). However, sensitivity compared to the RDT was similar in rural and urban populations. Similarly, participants with incomplete primary education performed the HIVST less well compared to the laboratory standard (80.6%; 95% CI: 62.5–92.5).

Performance was similar by sex of the user and when restricted to participants self-reporting HIV-negative or unknown HIV status (Table 4).

### Video recording sub-study

Of the 85 video-recorded participants (56 men and 29 women), only 4 were illiterate and a disproportionate number (48) came from urban health centre. (A comparison of videoed and non-videoed study sample by sex, location of data collection, and occupation is presented in Additional file 1: Table S6). There were 4 (4.7%) video recording sub-study participants who had reactive HIVST results. All 4 also tested HIV-positive on RDT and laboratory gold standard tests.

All testing steps up to placement into developer fluid were performed correctly by 75/85 (88.2%) of the participants. However, only 51 (60%) of participants read the manufacturer IFUs before the start of the test and 7/85 (8.2%) did not consult the IFU at all.

Specimen collection was the most difficult step with 78/85 (91.8%) managing to swab correctly. There was strong evidence of an association between literacy and being able to complete all steps of the test correctly (p = 0.004) (Additional file 1: Table S7.) Participants reported that the test was “very easy to do” (78/85, 91.8%) and most were confident that they had correctly interpreted their results (79/85, 92.9%). Participants self-reported fewer errors in completing the test than were observed. The most commonly self-reported error was spilling the developer fluid (Additional file 1: Tables S8–10).

## Discussion

In this study, the user-conducted user-read OraQuick® HIVST was 94.1% sensitive (95% CI: 90.2–96.7) and 99.7% specific (95% CI: 99.3–99.9) compared with the Zambian national RDT algorithm, and 87.5% sensitive (95% CI: 82.7–91.3) and 99.7% specific (95% CI: 99.4–99.9) compared with the laboratory reference standard. Although HIVST sensitivity was reasonable compared to the Zambian national RDT algorithm, both HIVST and the RDT algorithm had low sensitivity compared to our laboratory reference standard. Specificity of the HIVST was high when compared with both testing algorithms. Our laboratory reference standard algorithm used two 4^th^ generation combination antigen and antibody detection tests and HIV RNA testing in a fully accredited laboratory. We anticipated lower sensitivity due to the inherent limitations of antibody-based HIV tests, but the extent of difference is notable.

A recent systematic review and meta-analysis reviewed 25 studies, all of which measured concordance between the result from the self-tester and a professional tester [5], with only 15 studies calculating sensitivity and specificity against a reference standard, most commonly finger-stick RDT. The main conclusions were that specificity of oral fluid HIVST was generally high, being > 98% in 13/16 reports (some studies had more than one group), but that sensitivity was highly variable, being > 93% in only 9/16 reports presented in the review. Previous accuracy studies assessing HIVST in Africa have reported varied results, including higher sensitivities than we report here, but with much smaller sample sizes and limited number of HIV positive participants [13–15]. Reference standards have tended to use RDTs in routine practice [16, 17], although one Kenyan study using EIA for some samples found similar levels of sensitivity to that reported here, but again sample size was small [18]. Of note, the sensitivity of blood-based HIVST may be generally higher than HIVST using oral fluid, and additional research on the accuracy and usability of these blood-based tests will be useful [19]

Our laboratory reference standard was developed in consultation with HIV Prevention Trials Network (HPTN) Laboratory Central of the National Institutes of Health and used two 4^th^ generation tests (including antigen detection) and HIV RNA detection [20, 21]. Clinical performance was evaluated from the perspective of current “standard of care” RDT-based HIV testing, the near-universal approach in sub-Saharan Africa. The pattern of misclassification shown in Additional file 1: Table S5 is most consistent with low levels of circulating antibodies making the sensitivity of the whole blood assay lower. Plasma samples from the same participants do show higher sensitivity as would be expected when the additional limitations of fingerstick blood collection are removed.

The study also enables direct comparison of the RDT algorithm with the laboratory standard. Greater sensitivity was anticipated for our laboratory reference standard for reasons including a shorter “window period” with better performance in acute HIV infection due to detection of viral antigen and nucleic acid (RNA) in addition to host antibody response. However, with our study design acute HIV is unlikely for all but a few participants (two participants had detectable viral load, and many PLHIV in our sample were likely diagnosed and using ART). 4^th^ generation tests are also less affected by long-term ART. However, the RDT algorithm sensitivity of 93.4% against the laboratory reference was unexpectedly low and of potential regional relevance. Zambia uses a widely adopted combination of RDTs in their national algorithm: Alere Determine as the A1 (initial sensitive) test with Unigold A2 (confirmatory specific) test. While RDTs often perform very well, kit quality issues and user-errors may occur [21]. Stringent quality assurance systems and well selected testing algorithms aligned to WHO guidance are essential to optimizing performance. Finally, false-positive results have been reported for 4^th^ generation laboratory tests, and test performance for both RDTs and laboratory assays can vary in different global regions and the prevalence in the population being tested. Ensuring a verified national algorithm is implemented, and that no individual is diagnosed with HIV on a single assay, remains critical. Zambia is currently completing a verification study to update the national RDT-based algorithm in antenatal care settings with results anticipated in 2022 (Personal communication with C.C. Johnson, 13 January 2022).

Participants needed brief in-person demonstrations in addition to manufacturer IFUs to achieve the levels of accuracy reported here. Rural residents and those with incomplete primary education had lower test accuracy than urban participants and those with additional education. Importantly, many participants did not recognise the value of IFUs, with only 60.7% consulting them before starting to self-test. Programmes introducing HIVST, especially in rural settings, need to be aware that additional support is likely to be essential when HIVST is first introduced, and that linkage to additional confirmatory testing is required for a completed diagnosis.

HIVST can be conducted with the help of health workers (assisted) or by the individual, who can purchase the product over the counter in pharmacies or grocery stores (unassisted), using manufacturer IFU [1]. A systematic review and meta-analysis that included 21 studies, found that both assisted and unassisted HIVST resulted in an increase in partner-testing and were highly accept﻿able [22]. However, somewhat lower sensitivity was reported in unassisted versus assisted self-tests ranging from 92.9%-100% and 97.7%-97.9%, respectively.

HIVST is recommended by the World Health Organization (WHO) and has huge potential for scaling up testing and reaching population groups who had previously not been reached well by HTS programmes. While many users find HIVST to be very usable [23], implementers need to be mindful that self-testing is still a new concept in many situations. First-time users may be more comfortable conducting tests after a brief demonstration, videos, apps or on-demand additional instructions [13, 24, 25]. In these materials, implementers can also stress the importance of not testing while using ART, and provide information on linkage to HIV care or prevention services. In this study setting, mobile data and smartphone usage were low, so streaming video and other digital aids were not used. However, HIVST distribution programmes in South Africa and Zimbabwe have successfully used digital tools to support HIVST use [26, 27]. An assessment of users’ ability to perform and interpret an HIV self-test is also a requirement of the WHO pre-qualification process for test devices which closely follows the methods used in this study [28, 29]. To date there are now four WHO prequalified self-tests that have met the performance and usability standards, and the pipeline remains strong [30, 31].

Additionally, we found lower sensitivity compared with the laboratory algorithm among rural testers and those with lower levels of education. This is similar to findings from Zimbabwe where rural testers also struggled to follow instructions [8]. In West Africa, Tonen et al. also found lower levels of education and literacy affected the ability to correctly use blood-based HIVST [32, 33], and a South African study showed variable HIVST sensitivity within local populations [22]. We deliberately included both rural and urban participants because cognitive interviews had already shown more pronounced difficulty with HIVST in rural settings, reflecting both lower levels of literacy and also inexperience with commercially packaged products and the standard pictorial images used in IFUs [26].

Our video analysis, showing that many participants were not accustomed to procedures which require detailed study of instructions, has general relevance for self-testing beyond HIVST. Consumer literacy is different to absolute literacy, relating more to product availability in communities than individual factors. As more products with instructions for use become available, community literacy will grow. Techniques based on cognitive interviewing may allow implementers to rapidly identify communities where additional support is needed, potentially avoiding a period of low HIVST performance [26].

This study has several strengths, being a large random sample of the general population conducting HIVST in their own home and in a health facility, and inclusion of both RDT as well as a laboratory reference standard. Limitations include numbers of video recordings and use of demonstrations, necessitated by the unacceptable results from piloting reliance on manufacturer IFUs alone. Moreover, video-recorded participants gave consent and were fully aware they were observed by a health care worker and recorded while performing the self-test. We can therefore not exclude that the awareness of being observed changed the participants’ behaviour (the so-called Hawthorne effect) [34]. The study did not achieve full sample size, because of constraints on time for study recruitment. Finally, the study was conducted in 2016 prior to national or global self-testing guidelines and local registration and WHO prequalification of self-testing products. HIVST has been widely scaled-up in Zambia since the study and findings on usability and performance may continue to change over time.

## Conclusion

This study adds to the growing literature on HIVST, which challenges manufacturers, regulators and policy makers to ensure that all intended users, not just the better educated and most consumer-literate, can perform HIVST safely and accurately. We found that HIVST had acceptable sensitivity compared with laboratory testing or the Zambian national RDT algorithm only after an in-person demonstration was provided. We also found reduced sensitivity in rural populations and populations with lower educational attainment. Sustainable ways to provide additional support for self-testers should be part of HIVST introduction to disadvantaged communities, and should be considered for other self-care products. HIVST is a powerful tool in the fight against HIV and can reach previously untested individuals, but maximising impact requires correct use and interpretation [7].

We also report lower than anticipated sensitivity of the national RDT algorithm, which is of concern and underscores the need for ongoing monitoring and training as countries approach 95-95-95 targets. Repeat testing as determined by risk behaviour mitigates the impact of any one inaccurate result. As more countries scale up HIVST, the need for quality control for test kits and appropriate support for testers should be anticipated with early engagement of regulators and national reference laboratories [35, 36]. The lessons learned from this study on HIVST are critical for emerging diagnostic products for self-care.

## Supplementary Information

**Additional file 1: Table S1.** Demonstration Instructions. **Figure S1.** Laboratory reference testing algorithm for STAR clinical performance study. **Table S2.** Video Checklist. **Table S3.** Sensitivity analysis excluding known HIV+. **Table S4.** Sensitivity of RDT Vs Laboratory Reference Standard. **Table S5.** Testing details of participants with discordant RDT and laboratory algorithms. **Table S6.** Characteristics of respondents in CPS with video and CPS-only sub-samples. **Table S7.** Determinants of completing all steps correctly among video sub-sample. **Table S8.** Errors observed during video review. **Table S9.** User perceptions of HIVST (N=85). **Table S10.** User-reported errors in CPS with video and CPS-only sub-samples.

## Data Availability

The complete study protocol is available online at hivstar.lshtm.ac.uk, and anonymized data from the study is available for download upon application at datacompass.lshtm.ac.uk.

## Abbreviations

ART: Anti-retroviral therapy
CAP: College of American Pathologists
EDTA: Ethylenediaminetetraacetic acid
EIA: Enzyme immunoassay
EQA: External Quality Assurance
HIV: Human immunodeficiency virus
HIVST: HIV self-test
HPTN: HIV Prevention Trials Network
HTS: HIV testing services
IFU: Instructions for use
OFT: Oral fluid test
PLHIV: People living with HIV
RA: Research assistant
RDT: Rapid diagnostic test
STARD: Standards for Reporting Diagnostic Accuracy Studies
WHO: World Health Organization
