# Supplementary material for: Sensitivity and specificity of OraQuick® HIV self-test compared to a 4th generation laboratory reference standard algorithm in urban and rural Zambia
Source: BMC Infect Dis. 2022 May 25;22(Suppl 1):494. doi: 10.1186/s12879-022-07457-5 (PMC9134574; doi:10.1186/s12879-022-07457-5)
Supplement: Supplementary file 1 — Additional file 1: Table S1. Demonstration Instructions. Figure S1. Laboratory reference testing algorithm for STAR clinical performance study. Table S2. Video Checklist. Table S3. Sensitivity analysis excluding known HIV+. Table S4. Sensitivity of RDT Vs Laboratory Reference Standard. Table S5. Testing details of participants with discordant RDT and laboratory algorithms. Table S6. Characteristics of respondents in CPS with video and CPS-only sub-samples. Table S7. Determinants of completing all steps correctly among video sub-sample. Table S8. Errors observed during video review. Table S9. User perceptions of HIVST (N=85). Table S10. User-reported errors in CPS with video and CPS-only sub-samples. [file 12879_2022_7457_MOESM1_ESM.docx]

**Sensitivity and specificity of OraQuick® HIV self-test: a cross-sectional clinical performance study in intended users in urban and rural Zambia including a 4^th^ generation laboratory reference standard algorithm**

List of supplemental tables and figures

- Additional file 1: Table 1. Demonstration Instructions
- Additional file 1: Figure 1. Laboratory reference testing algorithm for STAR clinical performance study
- Additional file 1: Table 2. Video Checklist
- Additional file 1: Table 3. Sensitivity analysis excluding known HIV+
- Additional file 1: Table 4: Sensitivity of RDT Vs Laboratory Reference Standard
- Additional file 1: Table 5. Testing details of participants with discordant RDT and laboratory algorithms
- Additional file 1: Table 6. Characteristics of respondents in CPS with video and CPS-only sub-samples
- Additional file 1: table 7. Determinants of completing all steps correctly among video sub-sample
- Additional file 1: Table 8. Errors observed during video review
- Additional file 1: Table 9. User perceptions of HIVST (N=85)
- Additional file 1: Table 10. User-reported errors in CPS with video and CPS-only sub-samples

**Additional file 1: Table 1. Demonstration Instructions**

**1. This is the HIV ST kit.**

**2. When you open the kit, this is what you will find inside: Set of Instructions for Use.**

1. **Ensure you read through, both front and back, before you start doing the test.**
2. **Ensure you have something to use to TIME the test*.[watch/phone/timer]***
3. **This is the STAND; place it on a flat surface.**

**3. To open kit, find/see the opening points on the sides *[show sides];***

1. **Start with the side showing the picture of a bottle/tube.**
   - 1. **Remove tube/bottle &Open it *[show].***
     2. **Slide bottle/tube into the stand *[show].***
2. **Next open the other side to remove the test stick.**

**i. DO NOT use the small pack*[show desiccant]***

**ii. DO NOT touch the flat part of the test stick.**

1. **Note that the stick has a results window.**
2. **Two lines, next to C and T means a POSITIVE result= +.**
3. **One line next to C means a negative result= -**
4. **No line or reddish background means test did not work well. Need to REPEAT test with new kit =?**

**4 Using the test stick, swab along the upper gum ONCE and lower gum ONCE.**

**5 Place the test stick in the bottle/tube and WAIT for 20 MINUTES*[use your timer/watch/phone].***

**6 After twenty minutes, REMOVE stick from tube/bottle. READ the results**

1. **Two lines, next to C and T means a POSITIVE result= +.**
2. **One line next to C means a negative result= -**
3. **No line or reddish background means test did not work well. Need to REPEAT test with new kit =?**
4. **Put used test stick in small zip lock plastic *[provided* ]**

**7. Complete the SCQ [Record result and answer question]**

1. **Put SCQ in provided envelope**

**8. Put the rest of the used stuff in the other big zip lock plastic.**

**9. Bring back everything *[envelope & all plastics]* to me.**

**10. If there is anything that you will need to do the test, please come back and ask me.**

**11. If there is anything that you do not understand, please come back and ask me.**

**12. Please make sure you read through the instructions before you start doing the test.**

### **Additional file 1: Figure 1. Laboratory reference testing algorithm for STAR clinical performance study**

**Additional file 1: Table 2. Video Checklist**

|  | Y/N | comments |
| --- | --- | --- |
| **Reading the Instructions** |  |  |
| In- depth before testing |  |  |
| Just glanced at them before testing |  |  |
| Step-by-step |  |  |
| After specimen collection |  |  |
| not at all |  |  |
| **Opening the Packaging** |  |  |
| Opened (pouch) vial with picture first |  |  |
| Opened vial correctly |  |  |
| Placed the opened vial in the stand |  |  |
| Placed stand on an even surface |  |  |
| Opened the pouch with a picture of test device correctly |  |  |
| Touched the test device pad |  |  |
| Dipped the test device into the developer before testing |  |  |
| **Specimen collection** |  |  |
| Swab correctly (upper and lower gum) |  |  |
| Just swabbed lower or upper gum |  |  |
| Swabbed teeth |  |  |
| Licked on flat pad |  |  |
| Movement like a toothbrush |  |  |
| Sucked the test device |  |  |
| Swabbed several times |  |  |
| **Spilled the developer solution** |  |  |
| before specimen collection |  |  |
| after specimen collection |  |  |
| **Developing of results** | | |
| Put the flat pad of the test device into the vial correctly |  |  |
| Removed flat pad while waiting for the results |  |  |
| Wrote down start time/ set an alarm/ watch for 20 min |  |  |
| Read the results after 20 Min |  |  |
| Requested assistance |  |  |
| Unasked interference by health worker/camera men |  |  |
| The whole process is recorded (instructions-20min wait) |  |  |
|  | | |

**Additional file 1: Table 3. Sensitivity analysis excluding known HIV+**

|  | **Rapid Diagnostic test (RDT) Algorithm Result*** | | | **Laboratory Reference Standard Result**** | | |
| --- | --- | --- | --- | --- | --- | --- |
|  | **Positive** | **Negative** | **Sub-total*** | **Positive** | **Negative** | **Sub-total**** |
| **Participant-read reactive** | 185 | 8 | 193 | 186 | 7 | 193 |
| **Participant-read non-reactive** | 13 | 2304 | 2317 | 31 | 2286 | 2317 |
| **Sub-total** | 198 | 2312 | 2510 | 217 | 2293 | 2510 |
|  |  |  |  |  |  |  |
| **Agreement (%)** | 99.16 |  |  | 98.49 |  |  |
| **Cohen's kappa** | 0.9419 | 95% CI: 0.9171- 0.9666 |  | 0.8991 | 95% CI: 0.8677- 0.9311 |  |
| **Sensitivity (%, 95% CI)** | 93.4 | 89.0-96.5 |  | 85.7 | 80.3-90.1 |  |
| **Specificity (%, 95% CI)** | 99.7 | 99.3-99.9 |  | 99.7 | 99.4-99.9 |  |
|  |  |  |  |  |  |  |

* Excludes 9 OraQuick® results read as invalid by client , 5 clients with indeterminate RDT results, 3 clients missing RDT results, and 2 clients missing OraQuick® results (19 total)

** Excludes 9 OraQuick® results read as invalid by client , 8 clients missing laboratory results, and 2 clients missing laboratory and OraQuick® results (19 total)

**Additional file 1: Table 4: Sensitivity of RDT Vs Laboratory Reference Standard**

| **Agreement between nurse-administered RDT and Laboratory Reference Standard** | | |  |
| --- | --- | --- | --- |
|  |  |  |  |
|  | **Reference standard HIV- positive** | **Reference Standard HIV- negative** | **Sub-total*** |
| **RDT positive** | 241 | 0 | 241 |
| **RDT negative** | 17 | 2,295 | 2,312 |
| **Sub-total** | 258 | 2,295 | 2,553 |
|  |  |  |  |
| **Agreement (%)** | 99.34 |  |  |
| **Cohen's kappa** | 0.9630 | 95% CI: 0.9454, 0.9806 |  |
| **Sensitivity (%, 95% CI)** | 93.4 | 89.7-96.1 |  |
| **Specificity (%, 95% CI)** | 100 | 99.8-100 |  |
|  |  |  |  |
| * Excludes 6 self-reported ART users, 5 clients with indeterminate RDT results, 5 clients missing RDT results, and 10 clients missing lab results (21 total; 5 clients missing both RDT and laboratory result) | | | |

**Additional file 1: Table 5. Testing details of participants with discordant RDT and laboratory algorithms***

|  | **Field testing** | | **Laboratory testing on plasma** | | | | |
| --- | --- | --- | --- | --- | --- | --- | --- |
|  | **Self-test oral fluid** | **Field RDT algorithm fingerstick** | **Determine** | **Unigold** | **Abbott architect (Signal/cut-off ratio)** | **Biorad** | **Viral load** |
| 160162 | Non-reactive | Negative | Non-reactive | Non-reactive | Reactive (1.23) | Reactive | HIV-1 NOT DET |
| 160173 | Non-reactive | Negative | Faint Positive | Faint Positive | Reactive (5.83) | Reactive | HIV-1 NOT DET |
| 160191 | Non-reactive | Negative | Faint Positive | Faint Positive | Reactive (2.01) | Reactive | HIV-1 NOT DET |
| 160255 | Non-reactive | Negative | REACTIVE | Non-reactive | Reactive (1.30) | Reactive | HIV-1 NOT DET |
| 160322 | Non-reactive | Negative | Non-reactive | Non-reactive | Reactive (1.01) | Reactive | HIV-1 NOT DET |
| 160383 | Non-reactive | Negative | Non-reactive | Non-reactive | Reactive (1.25) | Reactive | HIV-1 NOT DET |
| 160384 | Non-reactive | Negative | Reactive | Faint Positive | Reactive (1.09) | Reactive | HIV-1 NOT DET |
| 160448 | Non-reactive | Negative | Faint Positive | Faint Positive | Reactive (7.29) | Reactive | HIV-1 NOT DET |
| 260511 | Non-reactive | Negative | Non-reactive | Faint Positive | Reactive (1.11) | Reactive | HIV-1 DET 2.56E05 Copies/ml(log 5.41) |
| 260633 | Non-reactive | Negative | Non-reactive | Faint Positive | Reactive (1.37) | Reactive | HIV-1 NOT DET |
| 261885 | Non-reactive | Negative | Reactive | Reactive | Reactive (30.76) | Reactive | HIV-1 DET 5.57E04 Copies/ml(log 4.75) |
| 261712 | Non-reactive | Negative | Faint Positive | Non-reactive | Reactive (1.12) | Reactive | HIV-1 NOT DET |
| 261983 | Non-reactive | Negative | Faint Positive | Non-reactive | Reactive (10.67) | Reactive | HIV-1 NOT DET |
| 262018 | Non-reactive | Negative | Faint Positive | Non-reactive | Reactive (3.36) | Reactive | HIV-1 NOT DET |
| 262019 | Non-reactive | Non-reactive | Non-reactive | Non-reactive | Reactive (1.23) | Reactive | HIV-1 NOT DET |
| 160723 | Non-reactive | Non-reactive | Non-reactive | Non-reactive | Reactive (1.50) | Reactive | HIV-1 NOT DET |
| 262105 | Non-reactive | Non-reactive | Faint Positive | Faint Positive | Reactive (3.81) | Reactive | HIV-1 NOT DET |

* No respondents were self-reported HIV-positive on ART

**Additional file 1: Table 6. Characteristics of respondents in CPS with video and CPS-only sub-samples**

|  |  | **CPS only** | | **Video sub-sample** | | **Total CPS sample** | |  |
| --- | --- | --- | --- | --- | --- | --- | --- | --- |
|  |  | **(N=2,489)** | | **(N=85)** | | **(N=2,574)** | |  |
|  |  | (n) | % | (n) | % | (n) | % | p-value* |
| Male |  | 989 | 39.7 | 56 | 65.9 | 1045 | 40.6 | <0.001 |
| Age in categories | |  |  |  |  |  |  |  |
|  | 15-17 yrs. | 107 | 4.3 | 1 | 1.2 | 108 | 4.2 | 0.528 |
|  | 18-24 yrs. | 990 | 39.8 | 37 | 43.5 | 1027 | 39.9 |  |
|  | 25-34 yrs. | 760 | 30.5 | 22 | 25.9 | 782 | 30.4 |  |
|  | 35-44 yrs. | 359 | 14.4 | 14 | 16.5 | 373 | 14.5 |  |
|  | 45-54 yrs. | 135 | 5.4 | 4 | 4.7 | 139 | 5.4 |  |
|  | 55 yrs. and older | 138 | 5.5 | 7 | 8.2 | 145 | 5.6 |  |
| Educational attainment | |  |  |  |  |  |  |  |
|  | Incomplete primary education | 207 | 8.5 | 4 | 4.7 | 211 | 8.4 | 0.375 |
|  | Complete primary education | 452 | 18.6 | 19 | 22.4 | 471 | 18.7 |  |
|  | Secondary or higher education | 1770 | 72.9 | 62 | 72.9 | 1832 | 72.9 |  |
| Able to read a newspaper or letter | | 2332 | 93.7 | 81 | 95.3 | 2413 | 93.7 | 0.549 |
| Occupation categories | |  |  |  |  |  |  |  |
|  | Agriculture, fishing, forestry | 318 | 12.8 | 18 | 21.2 | 336 | 13.1 | 0.012 |
|  | Other employment | 628 | 25.2 | 27 | 31.8 | 655 | 25.4 |  |
|  | Not employed | 1543 | 62 | 40 | 47.1 | 1583 | 61.5 |  |
| Data collection location | |  |  |  |  |  |  |  |
|  | Rural community | 584 | 23.5 | 29 | 34.1 | 613 | 23.8 | <0.001 |
|  | Urban community | 1030 | 41.4 | 8 | 9.4 | 1038 | 40.3 |  |
|  | Urban health facility | 875 | 35.2 | 48 | 56.5 | 923 | 35.9 |  |
| Language | |  |  |  |  |  |  |  |
|  | Bemba | 552 | 22.2 | 17 | 20 | 569 | 22.1 | 0.579 |
|  | Nyanja | 1596 | 64.1 | 58 | 68.2 | 1654 | 64.3 |  |
|  | Tonga | 147 | 5.9 | 2 | 2.4 | 149 | 5.8 |  |
|  | English | 26 | 1 | 1 | 1.2 | 27 | 1 |  |
|  | Other | 168 | 6.7 | 7 | 8.2 | 175 | 6.8 |  |

**Additional file 1: Table 7. Determinants of completing all steps correctly among video sub-sample**

|  |  | **At least one incorrect** | | **Completed all steps correctly** | | **Total video subsample*** | |  |
| --- | --- | --- | --- | --- | --- | --- | --- | --- |
|  |  | **(N=9)** | | **(N=75)** | | **(N=84)** | |  |
|  |  | (n) | % | (n) | % | (n) | % | p-value** |
| Male |  | 6 | 66.7 | 49 | 65.3 | 55 | 65.5 | 0.625 |
| Age in categories | |  |  |  |  |  |  |  |
|  | 15-17 yrs. | 0 | 0 | 1 | 1.3 | 1 | 1.2 | 0.807 |
|  | 18-24 yrs. | 4 | 44.4 | 33 | 44 | 37 | 44 |  |
|  | 25-34 yrs. | 2 | 22.2 | 20 | 26.7 | 22 | 26.2 |  |
|  | 35-44 yrs. | 1 | 11.1 | 12 | 16 | 13 | 15.5 |  |
|  | 45-54 yrs. | 1 | 11.1 | 3 | 4 | 4 | 4.8 |  |
|  | 55 yrs. and older | 1 | 11.1 | 6 | 8 | 7 | 8.3 |  |
| Educational attainment | |  |  |  |  |  |  |  |
|  | Incomplete primary education | 2 | 22.2 | 2 | 2.7 | 4 | 4.8 | 0.001 |
|  | Complete primary education | 5 | 55.6 | 13 | 17.3 | 18 | 21.4 |  |
|  | Secondary or higher education | 2 | 22.2 | 60 | 80 | 62 | 73.8 |  |
| Able to read a newspaper or letter | | 6 | 66.7 | 74 | 98.7 | 80 | 95.2 | 0.003 |
| Occupation categories | |  |  |  |  |  |  |  |
|  | Agriculture, fishing, forestry | 3 | 33.3 | 15 | 20 | 18 | 21.4 | 0.596 |
|  | Other employment | 2 | 22.2 | 25 | 33.3 | 27 | 32.1 |  |
|  | Not employed | 4 | 44.4 | 35 | 46.7 | 39 | 46.4 |  |
| Data collection location | |  |  |  |  |  |  |  |
|  | Rural community (Kanakantapa) | 5 | 55.6 | 24 | 32 | 29 | 34.5 | 0.262 |
|  | Urban community (Mtendere) | 1 | 11.1 | 7 | 9.3 | 8 | 9.5 |  |
|  | Urban health facility (Mtendere) | 3 | 33.3 | 44 | 58.7 | 47 | 56 |  |
| Language |  |  |  |  |  |  |  |  |
|  | Bemba | 1 | 11.1 | 16 | 21.3 | 17 | 20.2 | 0.723 |
|  | Nyanja | 8 | 88.9 | 49 | 65.3 | 57 | 67.9 |  |
|  | Tonga | 0 | 0 | 2 | 2.7 | 2 | 2.4 |  |
|  | English | 0 | 0 | 1 | 1.3 | 1 | 1.2 |  |
|  | Other | 0 | 0 | 7 | 9.3 | 7 | 8.3 |  |
| Previously tested for HIV | | 6 | 66.7 | 64 | 85.3 | 70 | 83.3 | 0.168 |
|  |  |  |  |  |  |  |  |  |
| * Excludes one respondent missing data on timer | | |  |  |  |  |  |  |
| ** P-values calculated using Fisher's exact test (two-sided) | | | |  |  |  |  |  |

**Additional file 1: Table 8. Errors observed during video review**

| Common user errors | n/N | % |
| --- | --- | --- |
| Test preparation |  |  |
| Did not read the instructions at all | 7/85 | 8.24 |
| Placed the cardboard stand on an uneven surface | 5/85 | 5.9 |
| Sample collection |  |  |
| Touched the pad of the test device  Did not swab correctly   - Swabbed teeth - Swabbed lips | 2/85  7/85  4/85  1/85 | 2.4  8.2  4.7  1.2 |
| Timing |  |  |
| Did not set an alarm | 16/84 | 19.0 |
| Did not wait 20 minutes | 1/16 | 6.25 |

**Additional file 1: Table 9. User perceptions of HIVST (N=85)**

|  | | (n) | % |
| --- | --- | --- | --- |
| How easy was the HIVST? | | | |
|  | Very easy | 78 | 91.8 |
|  | Somewhat easy | 5 | 5.9 |
|  | Somewhat difficult | 2 | 2.4 |
| Most difficult part of the test? | | | |
|  | Swabbing | 2 | 2.4 |
|  | Putting test device into the vial | 1 | 1.2 |
|  | Not touching the pad | 1 | 1.2 |
|  | Reading the result | 1 | 1.2 |
|  | All steps were easy | 80 | 94.7 |
| How easy was it to understand the IFU? | | | |
|  | Very easy | 74 | 87.1 |
|  | Somewhat easy | 10 | 11.8 |
|  | Somewhat difficult | 1 | 1.2 |
| Do you think the result was correct? | | | |
|  | Definitely | 79 | 92.9 |
|  | Probably | 5 | 5.9 |
|  | Probably not correct | 1 | 1.2 |

**Additional file 1: Table 10. User-reported errors in CPS with video and CPS-only sub-samples**

|  |  | **CPS only** | | **Video subsample** | | **Total CPS sample** | |  |
| --- | --- | --- | --- | --- | --- | --- | --- | --- |
|  |  | **(N=2,489)** | | **(N=85)** | | **(N=2,574)** | |  |
|  |  | (n) | % | (n) | % | (n) | % | p-value* |
| No errors | | 2472 | 99.4 | 82 | 96.5 | 2554 | 99.3 | 0.02 |
| Errors reported | |  |  |  |  |  |  |  |
|  | Spilled developer fluid | 10 | 0.4 | 1 | 1.2 | 11 | 0 |  |
|  | Touched the flat pad | 1 | 0 | 1 | 1.2 | 2 | 0.1 |  |
|  | Read the results before the time | 2 | 0.1 | 1 | 1.2 | 3 | 0.1 |  |
|  | Rubbed the wrong part of the mouth | 1 | 0 | 0 | 0 | 1 | 0 |  |
|  | Other error | 1 | 0 | 0 | 0 | 1 | 0 |  |
